# Sympathy Crying: Insights from Infrared Thermal Imaging on a Female Sample

**DOI:** 10.1371/journal.pone.0162749

**Published:** 2016-10-07

**Authors:** Stephanos Ioannou, Paul Morris, Samantha Terry, Marc Baker, Vittorio Gallese, Vasudevi Reddy

**Affiliations:** 1 Alfaisal University, Department of Physiological Sciences, College of Medicine, Riyadh, Kingdom of Saudi Arabia; 2 Department of Psychology-Centre for Situated Action and Communication, University of Portsmouth, Portsmouth, United Kingdom; 3 Parma University, Department of Neuroscience, Section of Human Physiology, Parma, Italy; 4 Institute of Philosophy, School of Advanced Study, University of London, London, United Kingdom; University of Bologna, ITALY

## Abstract

Sympathy crying is an odd and complex mixture of physiological and emotional phenomena. Standard psychophysiological theories of emotion cannot attribute crying to a single subdivision of the autonomic nervous system (ANS) and disagreement exists regarding the emotional origin of sympathy crying. The current experiment examines sympathy crying using functional thermal infrared imaging (FTII), a novel contactless measure of ANS activity. To induce crying female participants were given the choice to decide which film they wanted to cry to. Compared to baseline, temperature started increasing on the forehead, the peri-orbital region, the cheeks and the chin before crying and reached even higher temperatures during crying. The maxillary area showed the opposite pattern and a gradual temperature decrease was observed compared to baseline as a result of emotional sweating. The results suggest that tears of sympathy are part of a complex autonomic interaction between the sympathetic and the parasympathetic nervous systems, with the latter preceding the former. The emotional origin of the phenomenon seems to derive from subjective internal factors that relate to one’s personal experiences and attributes with tears arising in the form of catharses or as part of shared sadness.

## Introduction

Crying constitutes an important element of human emotional expression [1] and like blushing is unique to *homo sapiens* [2]. According to [3], various crying displays such as watery eyes, sobbing and weeping are part of an emotional intensity scale that is influenced by culture as well as individual trait variations. Functional distinctions in tear shedding were noted by [4] who separated crying in displays of suffering from “tender feelings”. Tears are seen and expressed in a variety of emotional contexts. Crying is first observed in the context of a simple response to pain or separation. However, during ontogeny, crying develops as a response to the distress of others in the form of sympathy [5–8].

Along with separation and loss, sympathy crying is among the most common causes of crying in adults [8]. Scientists [7] suggest that crying out of sympathy occurs when one *identifies with the victim* without this implying that the victim needs to cry as well. In fact, manifested signs of the victim’s suffering may not even be present. However, it is rather the attribution of cognitive and affective states by the observer to another person, centred on the evaluation of the sufferers’ situation, that elicit tears of sympathy [6]. This response arises in a variety of contexts whether it is joy coming after worry in the form of “catharsis” [2], or when it appears in a state of perceived helplessness, either as a part of the observer’s identification with the victim or their own realisation that nothing can be done to remedy a situation. Crying seems to serve a social function as theorists argue that tear shedding strengthens social bonds, sympathy, and comfort within a group [9–11]

Emotions seem to be “contagious” in nature and as brain imaging studies document the same viscero-motor and sensori-motor cortical regions are activated when witnessing an expressed emotion [12–16] or a given sensation [17]. Embodied simulation (ES) argues that people re-use their own mental states or cognitive processes represented through a bodily format to functionally attribute them to the state of others [6]. The above observations and psychological transactions on empathy, not only do they find ground through observations made with brain imaging studies but as it seems they are also exhibited through peripheral physiology. A phenomenon of autonomic synchronicity has been demonstrated where a mother shared a decrease of facial temperature with her child after it was exposed to a short-lived stressor [16].

There has been a debate about the extent to which different emotions have distinct patterns of autonomic activity and whether these emotions are associated with separate somatotopic maps and bodily-sensations [18]. Contemporary views argue that emotions are felt “somatically” with the autonomic nervous system (ANS) being an integral part of the emotional experience [19–22]. Studies on the physiological responses associated with crying have produced inconsistent results [23]. To be specific, some studies reported that during crying, electrodermal activity and heart rate increases as a result of sympathetic arousal [24–27] whereas others by using a variety of physiological measures suggest that crying is a phenomenon involving a complex mixture of sympathetic, parasympathetic and somatic arousal [28]. Support for the latter position comes from recent evidence indicating that crying causes an increase in respiratory sinus arrhythmia (RSA) associated with parasympathetic activity, emotional restoration and vagal engagement [1,29]. At a very basic level the ANS can be split into two competing subdivisions in which the parasympathetic division fosters non-emergency vegetative states, and the sympathetic division prepares the organism for “fight or flight” engagement strategies [24,30,31]. However, it is now well established that in many circumstances there is a far more subtle interplay between the parasympathetic and sympathetic nervous system. Crying seems to be an example where there is interaction between the two subdivisions of the ANS.

The actual act of crying is driven by the parasympathetic division of the ANS [32,33] as activation of the lacrimal glands is innervated solely by parasympathetic efferent fibers of the seventh cranial nerve. Nevertheless, for the arousal and exhibition of this phenomenon the competing subdivisions of the ANS either overlap for some period of time or there is a period of autonomic equilibrium that serves probably as an emotional transitory period from distress to physiological restoration. Both the arousal and the physiological recovery view fail to explain the functional social importance of crying [34–37]. Crying seems to have a beneficial psychophysiological effect as it reduces tension while achieving organismic homeostasis by replacing sympathetic with parasympathetic activity [23]. Although the exact temporal sequence of these physiological changes remains unclear [29]. It has been suggested that crying occurs shortly after the peak of an emotional episode [23,28].

‘Investigations of ANS responding in emotion have long been impeded by the exclusive use of “convenience measures,” such as HR and electrodermal activity, as sole indicators of the activation state of the organism’ [21] (p. 29). Functional Thermal Infrared Imaging (fTII) is a relatively new measure in psychophysiology where changes of the somatic thermal print of the face are taken to be a function of ANS activity [38,39]. The emitted thermal print is affected by parameters such as subcutaneous vasoconstriction or vasodilation [40–42], blood flow [43], perspiration [44], muscular activity [40,45], heart rate [46], and metabolic breathing patters [47]. The reliability of fTII as a physiological tool has been proven by simultaneous GSR recordings [48,49,50] as well as laser Doppler flowmetry [51]. Nevertheless, little is know about the involvement of facial temperature on different emotional states. Thermal variability of different region of the face could one day prove to be a measure in which internal states and emotions could be better understood since a variety of physiological reaction are depicted on the face [38]. Moreover unlike other physiological measures thermal imaging is an easily accessible physiological measure of emotional reactivity. Foundation work is key for the development of future experimental protocols that could be used as potential diagnostic measures of psychopathology and early screening tools of developmental disorders.

The majority of studies that examined crying in a sad context have used films to elicit this behavior [28], [29]. However, in the case of [28] only 33 out of 117 individuals cried. This is not surprising since crying as any other emotion is a subjective experience; therefore unless an individual shares a common experience with the sufferer then it is hard to sympathize with the situation [5–8], [52]. By taking these aspects into consideration the current study adopted a revised model to elicit crying. Participants were asked to choose prior to the experiment the film and the scene from the film that was most likely to cause them to cry. Physiological observations on crying have so far described this phenomenon to be associated with a heart rate decrease, and increases in RSA and skin conductance. Although cardiovascular measures suggest an association between vagal engagement and parasympathetic activity, increases in skin conductance tell a different story. Sweat secretion of affective nature originate mainly on the axillae, palm and soles of the feet mainly controlled by sympathetic efferent fibers [24,25,27]. Recent studies have shown that the thermal print of the upper lip or peri-nasal region in response to a startle stimulus shares a strong positive correlation with the GSR signal obtained on the palm [44,49]. Therefore by monitoring wirelessly from a distance the upper lip can provide information about sympathetic arousal.

Based on previous findings/literature it is expected that crying is going to be accompanied by an increase in temperature as a result of parasympathetic activity and physiological restoration [1,29]. Also, it is predicted that perspiration pores associated with a temperature decrese are going to be observed prior of crying on the maxillary area [44,49]. Thermal signs of physiological arousal are going to be extracted on six facial regions of interest (ROI) the maxillary, nose, cheek, chin, peri-orbital region and forehead based on the anatomical model provided by [38]. The results should provide detailed information about the temporal pattern of sympathetic and parasympathetic activity associated with crying. This information will enhance our understanding about the role of the sympathetic and parasympathetic in the expression of emotions.

## Methods & Materials

### Ethics

Experimental procedures were in line with the declaration of Helsinki and the code of Human research ethics of the British Psychological Society. Ethical approval was given by the Research Ethics Committee of the University of Portsmouth, Department of Psychology. All Participants were informed about the scope of the study and have all given their written consent to participate in the study. The individual in this manuscript has given written informed consent (as outlined in PLOS consent form) to publish these case details

### Design

A 3 x 13 fully repeated measures factorial design was employed. The within-subjects factors were period (baseline, pre- crying and crying) and 13 levels of time within each period.

### Participants

Thirteen female students, from a range of cultural backgrounds with an age range of 20 to 23 years (*M*_*age*_ = 21.23, *SD* = .93) took part in the study. Recruitment was performed using social networking sites and the University of Portsmouth recruitment database. Being easily moved to tears when watching sad films was the central criterion for participation. In order to improve the reliability of the physiological observation, consumption of vasoactive substances (nicotine, caffeine, alcohol) for at least 3 hours prior of participation was prohibited. All participants were advised not to use a dense layer of make-up during the day of the study.

### Stimulus Materials

Prior to participation in the study, all individuals identified an emotionally arousing film that would be used on the day of the experiment. Furthermore, participants identified the section of the film that they found most emotionally arousing. In the experiment, participants viewed the film starting 15 minutes prior to the relevant scene. Approximately thirty minutes of the film was viewed in total.

### Procedure

The study was conducted in a sound attenuated, climatically controlled psychophysiological laboratory. Participants were asked to comfortably sit on a chair, focus on their breathing and relax in order for skin baseline measures to be recorded [51,53]. After 5 minutes the temperature of the nose was monitored continuously by placing a circular extraction point on the nasal tip with a radius of 5 mm. The nose was selected because previous thermal imaging studies have found it to be the most reliable indicator of physiological arousal [38,45]. Once the temperature of the nose stabilised and did not fluctuate by more than ± .1°C for a period of 60 seconds then it was considered that the pseudo-baseline was established. The relevant films were then presented to the participants and were instructed to refrain from drying out their tears. Following the presentation of the film participants were given approximately five minutes to relax and regain composure. A questionnaire was then administered regarding their experience. Finally chocolates were given to the participant to cheer them up.

### Data Acquisition

Physiological recordings were performed with a Guide Infrared TP8 camera (ThermoPro™) with an uncooled FPA microbolometer (384 × 288 pixels), temperature sensitivity of 0.08 K, an accuracy of ± 1°C. To record facial skin temperature the camera was placed 1 meter away from the participant, in a direct angle with the participants’ face and was automatically calibrated and manually fixated on the individual’s face. The sampling rate was set at 30Hz and recordings took place in a thermally isolated experimental room with normal temperature 20–21°C, 60–65% humidity, and with no direct sunlight, ventilation or airflow. For the recordings the camera was calibrated daily between *T*_min_ = 30°C and *T*_max_ = 38°C with an external black body. Thermal data were saved on an external hard drive (buffalo, mini station). In addition, a JVC Everio GZ-HM30BEK video camera was used with a frame rate also set at 50Hz placed next to the thermal camera again at a 1 meter distance from the individuals face.

### Questionnaire

To examine the emotional experience of crying four questions were given for participants to answer. Three items used a 5 point Likert-type scale where 1 = Strongly Disagree to 5 = Strongly Agree. The three items were (a) ‘I felt really sad during the film’ (b)’ I really enjoyed the film’ (c) ‘The sadness I experience during the film is very different from the sadness I experience in everyday life’. The final question was an open ended question exploring other emotions they may have experience during the film. (d) Can you describe in more detail on how you were feeling during crying at the film, other than feeling sad.

### Thermal Data Analyses

Prior to analyses both thermal and behavioural videos were synchronized in order to represent the same point in time. The infrared video was split into three segments a) baseline b) pre-crying and c) crying. Data was extracted every five seconds and lasted one minute for the baseline and pre-crying phase. For each minute 13 frames were extracted (.00, .05, .10, .15, .20, .25, .30, .35, .40, .45, .50, .55, 01.00) all homogeneous in angle with the direction of the thermal recordings as variability in angle can induce considerable noise in the data sample [38]. The pre-crying phase was defined as the second that preceded the first tear and was defined by two independent raters. A Cohen’s Kappa was conducted on 10% of the sample and an acceptable level of reliability was observed (.86). For the reliability analyses 2 participants were included in all phases of the experiment (Baseline, Pre-Crying, Crying). From the 13 participants only 11 cried and these were the ones that were included in the main analyses. The crying phase lasted approximately from 3–4 minutes. Temperature values for the crying phase were the initial minute of the crying phase. The minute prior to the onset of crying was categorised as the pre crying period. Crying was not characterised by observable signs of lacrimal gland activation such as watery eyes, sobbing, and eye wiping along with sniffing. The baseline was established at the beginning of the experiment and lasted in total 60 seconds. Temperature was extracted from 6 regions of the face such as the forehead, peri-orbital region, nasal tip, maxillary area, the cheeks as well as the chin. The placement and shape of the regions of interest was as defined by [38] (p. 5). Nevertheless because the trail of tears obscured the region of interest, tracking of thermal changes was performed closer to the nasal region in the junction of the maxillary and facial artery using a circular ROI. Extraction of data was anatomically consistent throughout the experimental condition.

Thermal data extraction was performed manually with the software LaunchGuide IrAnalyser by Wuhan Infrared Technology (http://www.guide-infrared.com).

## Results

### Reliability

To examine inter-rater reliability 20% of the analyzed frames were selected. Prior to comparing the two data sets the average degree of change from one phase to the next was calculated. This was performed in order to eliminate differences in the size of the regions of interest selected by each independent rater (Table 1). An independent sample t-test showed that there was no significant difference (p > .05 [two tailed]) between the scores of rater 1 (M = .24, *SD* = .40) and rater 2 (M = .25, *SD* = .40) *t* (46) = .98, *p* = .98. The magnitude of the mean differences (= -.003, 95% CI -.236 to .230) was very small (eta squared = .0005). In addition a Spearman rho showed a large, positive, significant correlation between the scores of the raters, *rho (24)* = .99, *p* < .0001.

**Table 1:** The degree of temperature change from one condition to another based on the coding of two independent raters.

Contrast	Region	Rater 1	Rater 2	Rater 1	Rater 2
Pre-Cry vs. Baseline	Forehead	.15	.16	.55	.55
	Peri-orbital	.40	.40	.10	.10
	Nose	.23	.22	-1.22	-1.22
	Chin	-.07	-.07	.55	.56
	Maxillary	-.06	-.07	-.09	-.08
	Cheek	.44	.45	.97	.95
Crying vs. Pre-Cry	Forehead	.43	.43	.27	.29
	Peri-orbital	.22	.25	.18	.17
	Nose	.50	.45	.66	.66
	Chin	.60	.60	.05	.05
	Maxillary	.35	.35	.34	.40
	Cheek	.16	.16	.27	.29

### Individual Region Analyses

For each region of interest a 3 X 13 repeated measures ANOVA was conducted (three conditions: Baseline, Pre-crying, Crying X 13 (0.05s) time periods for each condition).

A significant main effect of condition was found for all regions of interest with large effect sizes (Table 2).

**Table 2:** Main effect analyses according to condition for each region of interest.*

Region of interest	F value	df	p	η_p_^2^
**Forehead**	18.37	1.24,12.37	< .001	.65
**Maxillary**	81.11	1.13, 11.26	< .01	.89
**Cheek**	28.34	1.64,16.44	< .001	.74
**Chin**	8.67	1.32, 31.23	< .01	.46
**Nose**	7.06	1.24,12.40	< .05	.41
**Peri-orbital**	4.39	1.73,17.36	< .05	.30

* df subject to Greenhouse Geisser where appropriate

**Fig 1:**
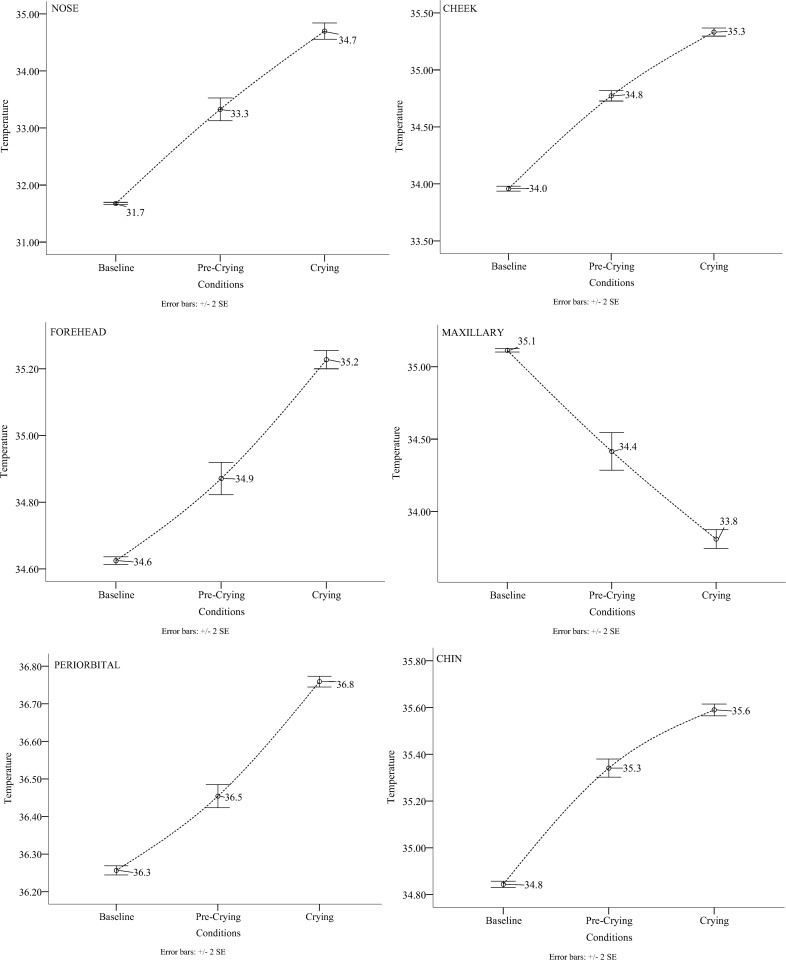
Line graph: Development of temperature for each region of interest according to condition.

**Fig 2:**
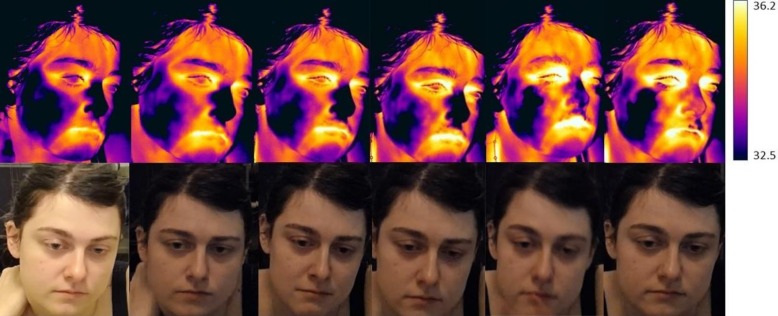
Thermo-grams: Development of temperature throughout each condition (two pictures are provided for baseline, pre-crying and crying).

The pattern of means was the same for the peri-orbital region, the forehead, the nose, the cheeks and the chin; the baseline period had the lowest mean temperature, the pre crying period the next lowest mean temperature and the crying period the highest mean temperature. The maxillary area however followed a different pattern in which the baseline had the highest temperature followed by the pre-crying temperature and then the crying temperature (Table 3) (Figs 1 and 2) (S1 Video).

**Table 3:** Means and Standard Deviations of Regions of Interest as a Function of Condition.*

Region	Condition	M	SD
**Forehead**	Baseline a	34.62	1.48
	Pre-Crying b	34.87	1.39
	Crying c	35.23	1.30
**Peri-orbital**	Baseline a	36.27	0.52
	Pre-Crying a	36.45	0.61
	Crying a	36.76	0.45
**Nose**	Baseline a	31.68	4.03
	Pre-Crying ab	33.33	3.08
	Crying 1 b	34.70	2.28
**Chin**	Baseline a	34.84	0.90
	Pre-Crying b	35.34	0.71
	Crying b	35.59	0.78
**Maxillary**	Baseline 1 a	35.11	0.96
	Pre-Crying b	34.41	1.00
	Crying c	33.81	1.09
**Cheek**	Baseline a	33.96	1.09
	Pre-Crying ab	34.77	1.06
	Crying b	35.33	0.95

*Means that do not share a subscript are significantly different from one another (multiple comparisons used Sidak adjustment)

A main effect of time was observed for the maxillary area *F* (2.00, 20.04) = 40.90, *p* < .001, η_*p*_^*2*^ = .80 and the nose *F* (1.08, 10.80) = 6.65, *p* = .02, η_*p*_^*2*^ = .40. Post-hoc comparisons revealed no differences between means although the arithmetic trend was for an increase as a function of time (Table 4). There was no main effect of time observed for the forehead, peri-orbital, chin and cheek regions of interest.

**Table 4:** Mean Temperature Values for the Nose and Maxillary Area According to Time.

Time	Nose-M	Nose-SD	Maxil.-M	Maxil.-SD
0	32.92	3.56	34.61	1.10
0.05	32.97	3.56	34.58	1.10
0.1	33.02	3.55	34.56	1.10
0.15	33.08	3.53	34.55	1.12
0.2	33.14	3.53	34.50	1.15
0.25	33.18	3.50	34.50	1.15
0.3	33.20	3.48	34.48	1.15
0.35	33.24	3.43	34.44	1.16
0.4	33.30	3.44	34.40	1.16
0.45	33.36	3.43	34.34	1.20
0.5	33.45	3.39	34.30	1.22
0.55	33.55	3.42	34.28	1.21

The only region of interest that revealed an interaction effect was the maxillary area, *F* (2.90, 29.02) = 10.93, *p* < .001, η_*p*_^*2*^ = .52. There was no change in temperature as a function of time in the baseline condition whereas temperature decreased as a function of time in pre-crying and crying. Analyses of linear trend revealed that the decrease in temperature as a function of time was significant with a large effect size for the pre-crying condition, *F* (1,10) = 8.75, *p* = .01, η_*p*_^*2*^ = .78 and the crying condition *F* (1,10) = 29.74, *p* = .00, η_*p*_^*2*^ = .75

### Questionnaire Responses

One sample t-tests were conducted between the participants’ responses and the midline value of the Likert-scale in order to examine if scores exceeded the median Likert value. For all three questions the participants mean score was significantly higher than the mid point of the Likert scale (Table 5).

**Table 5:** Mean scores and standard deviations for participant’s answers to each of the three questions according to their experience while watching the film.

	t(df)	p	Mean	SD
**Real Sadness**	8.95(12)	.001	4.61	.65
**Enjoyed the film**	4.50(12)	.001	4.08	.86
**Other type of sadness**	2.99(12)	.011	3.77	.93

### Open ended questions-Grounded Theory

To further characterise and understand the affective nature of crying and the psychological reason that lead to the arousal of this physiological phenomenon grounded theory was used following open coding. Three thematic categories arose by labelling the participants’ responses Sympathy & Empathy, embodied simulation and emotional contagion (Table 6).

**Table 6:** Representation of the coding performed for each participant according to thematic group.

**Part.**		**Theme: Empathy & Sympathy**
**P1**		. . .“Empathize”
**P2**		. . .“Sympathetic towards the family”
**P3**		. . .“Sympathy for the family”
**P4**		. . .“Compassionate”
**P5**		. . .“Empathy”
**P6**		. . .“Felt sorry for Simba”
**P7**		
**P8**		
**P9**		. . .“Empathetic towards the situation”
**P10**		
**P11**		
		**Theme: Embodied Simulation**
**P1**		. . .“Reflective”. . .“Applied to something that happened in my life”. . .“In their shoes”. . .“Brought back old emotions”
**P2**		. . .“Hard to imagine how hard it was”
**P3**		. . .“How I would feel if it was my dog”
**P4**		
**P5**		. . .“Could put myself in their shoes” Imagined it was my family”
**P6**		
**P7**		. . .“Put film narrative to own life”. . .“What would I do in that situation”
**P8**		. . .“Remembering similar experience”. . .“Losing family member due to cancer”
**P9**		
**P10**		. . .“Relate to own personal life–reminds me of nans death “. . .“means a lot to me”
**P11**		. . .“Put film into own situation-real life”
		**Theme: Emotions**
**P1**		. . .“Felt refreshed”. . . .”Happy memories”
**P2**		. . .“Sad”. . .“Film ‘touched’me-moved me”. . .“Emotionally sad”
**P3**		. . .“Moving”. . .“Distressing”
**P4**		. . .“Upsetting”
**P5**		. . .“Emotional”. . .“Happy they were reunited”
**P6**		. . . “Angry at Scar”
**P7**		
**P8**		. . .“Upset”. . .“Overwhelmed with sadness”
**P9**		
**P10**		. . .“Emotional film”
**P11**		

#### Sympathy

The theme of sympathy and empathy seemed to arise in 63.6% of participants’ responses. Participants not only used the actual words sympathy and empathy to describe the reason why they were crying but they also directed this social emotional experience to the sufferers and the situation in which they were implicated in.

#### Embodied simulation

The theme of embodied simulation arose in 72.7% of the discourses. The wording used under this thematic category was in the domain of personal experience, memory recall, self-reflection and mentalization. This makes it possible to speculate that those who cried when watching their chosen film, may have done so due to the fact that the characters of the movie were experiencing something very similar to what they had been through. Thus it was very easy for them to re-live emotionally the movie scenario.

#### Emotions

Different types of emotion seem to have arisen during the crying experience prevalent in 72.7% of the responses. The participants have mentioned that they were moved by the movie as well as that they felt refreshed and happy. In addition they reported also negative emotions such as distress, anger as well as well as sadness.

## Discussion

The current study was designed to study the thermal print of a crying episode as well as characterize the physiological nature of the tear reflex. The experimental protocol successfully induced crying in 11 individuals. For all participants temperature increased from baseline, to pre crying, to crying in all facial areas except the maxilliary area. In contrast temperature fell on the maxillary area. No significant temperature change was observed during baseline and temperature remained satisfactorily constant (± .1). The responses given by the participants to the three questions clearly indicated that although they felt sad during the film they really enjoyed it and that the sadness experienced during the film was in fact very different from the sadness experienced in everyday life. Finally crying seemed to arise as part of the participant’s ability to put “themselves in other’s shoes”, with the assumption that they either re-lived the same situation before or could imagine living the story depicted in the movie.

Temperature on five of the regions of interest rose substantially compared to baseline. In fact all regions of interest had a rise above .5°C and in the case of the nose above 3°C. The size of change on the maxillary area was also substantial; with a fall of more than 1°C. The above observations are the largest documented temperature changes in the psychophysiology of emotions. Cardiovascualar changes are believed to be responsible for the temperature rise of the face since [28] observed a heart rate increase in participants during crying. Temperature rise is related to blood flow increase to the surface of the skin. It is unlikely that subcutaneous vascular constriction has occurred since the nose did not show any decrease in temperature. Literature on the topic suggests that the nose is the most reliable indicator of distress and negative emotional arousal. Studies that have been conducted on humans [52] and non-human primates [45] have observed a temperature decrease on the nose during negative emotional displays; even when these displays were related to a heart rate increase [21]. Scientific findings by [54] suggest that the temperature decrease observed on the tip of the nose is related to the sympathetic division of the autonomic nervous system. Chimpanzees that watched a video of conspecifics fighting had lower heart rate variability an indication of vagal withdrawal and parasympathetic deactivation. On the contrary increases in temperature similar to the ones obtained in the current study have been reported in studies of social interaction. A temperature increase was observed when children were apologizing to the experimenter after breaking a toy [52], during direct gaze and intrusions of intimate space by a stranger [55] as well as interpersonal contact in various body parts through a handheld device [42]. Lastly whereas other regions of the face documented an increase, the maxillary area had a temperature decrease starting prior of crying. This phenomenon is related to sympathetic arousal and the activation of perspiration pores [44,49,56]. This observation is consistent with increases in skin conductance observed on the palms of participants during crying [26,28]. It seems highly likely that there is significant autonomic arousal associated with crying, however, the precise temporal relationship between crying and autonomic arousal remains unclear.

Close and open-ended questions have provided insight into the feelings that lead to crying. Associated with the film were negative emotions such as sadness, anger, and distress but many participants stated that the felt sensation was “different” to real life. Despite the fact that the participants reported experiencing negative emotions, most indicated that they actually enjoyed the film. The open-ended questions did provide some explanation into this emotional paradox. Participants made statements about events in the film such as “happy they were re-united” and also commented that they “felt refreshed”. Such reports are consistent with [2] explanation that postulated that sympathy crying might result as a form of joy after worry. Psychoanalytic interpretations regard crying as an act of catharsis, which triggers the release of tension and anxiety “bringing to the surface”, desires, feelings, thoughts and past memories into conscious awareness. Moreover according to the participants’ responses, crying arose because of the fact that they could relate to the content of the movie either through their own personal experiences (memory) or by mentally re-living the situation (imagination). Both explanations require the cognitive capacity to imagine themselves in the shoes of another. Embodied simulation incorporates the above explanations as participants experienced the portrayed negative cognitive and affective states of the actors in the sad film, by re-living the situation or by the recalling from memory a familiar incident [6]. Furthermore the participants have labeled their crying experience as sympathetic [8] or empathetic [5–7] giving a social component to the overall experience of crying.

The current study has managed to successfully induce crying in 11 out of 13 individuals and despite the limited number of participants temperature tendencies were consistent across all crying and pre-crying phases. Selecting participants who cry easily and allowing them to choose their own preferred sad film were key aspect of the methodology. Nevertheless we do recognise that the non-uniformity of the independent variable may be considered a significant flaw in the design of the study. No report to date has managed to have such a high crying rate among participants simply because one cannot sympathise to the extent of tearing if their life experiences do not remotely resemble those of others. In addition due to the contact-less nature of thermal imaging there was no interference with the experimental protocol or the natural exhibition of crying by the participant. Moreover the obtained results shed light to the controversy that exists about the biologically birth of the nasal temperature, argued to be the result of metabolic breathing patterns [16,38,52,57]. For example both fear and crying sadness are associated with a respiration rate increase [21] and one would generally expect (if the above argument was correct) for the temperature of the nose to decrease. The current study observed the opposite effect. In fact temperature of the nose was rising during crying. Thus it is rather unlikely that any significant changes related to the phenomenon of temperature decrease [45,48] or in this case rise to be related to breathing artifacts.

Despite the overall robust effect throughout the experiment, future studies on sympathy crying could benefit from the following aspects. A larger sample size as well as the inclusion of the opposite sex would make results more representative of the general population. Moreover although manual extraction allows more control over data extraction is indeed time consuming and for the current experiment it was not optimal. In experiments that entail stimuli presentation through a screen or in which the participants are relatively static, automatic tracking technology and extraction could make things very natural and the temporal as well as spatial resolution very fine. This can be achieved through FLIR Research IR software.

## Conclusions

Crying seems to be a distinctive physiological phenomenon. Previous and current research findings suggest that a blend of autonomic engagement occurs during crying. The tear reflex although associated with the parasympathetic division of the ANS, paradoxically it is also accompanied by an increase in heart rate and affective sweating. Although these aspects of heart rate and perspiration do belong to the sympathetic division of the ANS thermo-grams suggest that no sub-cutaneous vascular constriction occurred during this phenomenon as temperature should have been decreasing. This observation lets us conclude that a full-blown sympathetic episode did not arise and crying, as it seems does not have an isolated physiological birth. In extent crying arises not only to serve a physiological restorative function but as self-reports suggest to alleviate the mood of an individual after a sad episode. Furthermore, for crying to arise a mental bond between the observer and the sufferer needs to occur either in the form of a memory recall or as an imaginary representation of the event. Overall crying and particularly tears of sympathy work in a social content and have a high communicative element signaling to the sufferer and to the group that not only emotions are inter-individually shared but also that social bonds within the group are strong and work as a unified ‘soma’.

## Supporting Information

S1 Video The video clip shows in real time how temperature was developing across phases. Perspiration on the upper lip can be observed during pre-crying that intensifies and becomes more visually evident during crying.
(MOV)
